# Anti-PD-1/L1 antibody plus anti-VEGF antibody *vs.* plus VEGFR-targeted TKI as first-line therapy for unresectable hepatocellular carcinoma: a network meta-analysis

**DOI:** 10.37349/etat.2024.00236

**Published:** 2024-06-17

**Authors:** Yiwen Zhou, Jingjing Li, Jieer Ying

**Affiliations:** Sun Yat-Sen University Cancer Center, China; ^1^The Second School of Clinical Medicine, Zhejiang Chinese Medical University, Hangzhou 310053, Zhejiang, China; ^2^Department of Hepato-Pancreato-Biliary & Gastric Medical Oncology, Zhejiang Cancer Hospital, Hangzhou Institute of Medicine (HIM), Chinese Academy of Sciences, Hangzhou 310022, Zhejiang, China; ^3^Postgraduate training base Alliance of Wenzhou Medical University (Zhejiang Cancer Hospital), Hangzhou 310022, Zhejiang, China

**Keywords:** Hepatocellular carcinoma, anti-programmed death 1/ligand-1 antibody, anti-vascular endothelial growth factor antibody, tyrosine kinase inhibitor, network meta-analysis

## Abstract

**Background:**

This article is based on our previous research, which was presented at the 2023 ASCO Annual Meeting I and published in *Journal of Clinical Oncology* as Conference Abstract (*JCO*. 2023;41:e16148. doi: 10.1200/JCO.2023.41.16_suppl.e16148). Both anti-programmed death 1/ligand-1 (PD-1/L1) antibody + anti-vascular endothelial growth factor (VEGF) antibody (A + A) and anti-PD-1/L1 antibody + VEGF receptor (VEGFR)-targeted tyrosine kinase inhibitor (A + T) are effective first-line therapies for unresectable hepatocellular carcinoma. However, there lacks evidence from head-to-head comparisons between these two treatments. We conducted a network meta-analysis on the efficacy and safety of them.

**Methods:**

After a rigorous literature research, 6 phase III trials were identified for the final analysis, including IMbrave150, ORIENT-32, COSMIC-312, CARES-310, LEAP-002, and REFLECT. The experiments were classified into three groups: A + A, A + T, and intermediate reference group. The primary endpoint was overall survival (OS), and secondary endpoints included progression-free survival (PFS), objective response rate (ORR), and incidence of treatment-related adverse events (TRAEs). Hazard ratio (HR) with 95% confidence intervals (CI) for OS and PFS, odds ratio (OR) for ORR, and relative risk (RR) for all grade and grade ≥3 TRAEs were calculated. Under Bayesian framework, the meta-analysis was conducted using sorafenib as intermediate reference.

**Results:**

With the rank probability of 96%, A + A showed the greatest reduction in the risk of death, without significant difference from A + T (HR: 0.82, 95% CI: 0.65–1.04). A + T showed the greatest effect in prolonging PFS and improving ORR with the rank probability of 77%, but there were no statistical differences with A + A. A + A was safer than A + T in terms of all grade of TRAEs (RR: 0.91, 95% CI: 0.82–1.00) and particularly in those grade ≥3 (RR: 0.65, 95% CI: 0.54–0.77).

**Conclusions:**

A + A had the greatest probability of delivering the longest OS, while A + T was correlated with larger PFS benefits at the cost of a lower safety rate.

## Introduction

According to data from the GLOBOCAN 2020 database, liver cancer is a pronounced health burden worldwide, ranking sixth in incidence (4.7%) and third in mortality (8.3%) among all cancers [1]. Hepatocellular carcinoma (HCC) accounts for 75–85% of primary liver cancer cases [2]. Due to difficulties in early diagnosis, most HCC patients are diagnosed at an advanced stage and miss opportunities for radical treatment, thus effective systematic treatment is in high demand for them [3].

According to the results from the SHARP [4] and sorafenib (SOR) Asia-Pacific trials [5], SOR was the only targeted therapy allowed for HCC patients from 2007 until 2018, which significantly prolonged patients’ overall survival (OS). During that decade, many other compounds, including erlotinib, brivanib, sunitinib, linifanib, and everolimus, were tested in the first-line treatment of HCC, but they did not show superiority or noninferiority to SOR [6]. In 2018, lenvatinib (LEN) was found to be non-inferior to SOR and represented a long-awaited alternative option to SOR for the first-line systemic treatment of patients with unresectable HCC (uHCC) [7]. In recent years, clinical trials of a variety of combination regimens have generated mixed results. The results of IMbrave150 led to the largest change in the treatment landscape of uHCC, which showed that the combination of atezolizumab and bevacizumab was significantly superior to SOR with an improved median OS and comparable rates of ≥ 3 grade adverse events [8]. The results of the ORIENT-32 were also positive for OS and progression-free survival (PFS) [9]. With the combination of camrelizumab and apatinib, CARES-310 observed the longest OS (22.1 months) among all phase III trials for uHCC in 2023 [10]. However, the LEAP-002 trial showed that pembrolizumab plus LEN did not meet its dual primary endpoints of OS and PFS as a first-line treatment for patients with uHCC [11]. Similarly, treatment with atezolizumab with cabozantinib in COSMIC-312 significantly improved PFS compared to SOR, but it did not reach statistical significance for OS [12].

Based on the above studies, immunotherapy combined with targeted therapy is a widely used first-line therapy for uHCC and can be roughly divided into two types: anti-programmed death 1/ligand-1 (anti-PD-1/L1) antibody combined with vascular endothelial growth factor (VEGF)-antibody and anti-PD-1/L1 antibody combined with VEGF receptor (VEGFR)-targeted tyrosine kinase inhibitor (TKI) [13]. However, the choice of anti-PD-1/L1 antibody + VEGF-antibody (A + A) or anti-PD-1/L1 antibody + VEGFR-targeted TKI (A + T) for the first-line treatment of uHCC is a practical problem and a challenge for clinicians. Due to the absence of head-to-head clinical trials, we conducted a network meta-analysis (NMA) to indirectly compare the efficacy and safety between A + A and A + T.

## Materials and methods

### Search strategy and selection criteria

We conducted a NMA to indirectly compare the two different combination therapies: anti-PD-1/L1 antibody plus VEGF-antibody *vs.* anti-PD-1/L1 antibody plus VEGFR-targeted TKI. We specified that the interventions in the trial included any anti-PD-1/L1 antibody combined with any kind of VEGF antibody or combined with any kind of VEGFR-targeted TKI. Additionally, to satisfy the establishment of indirect comparison, we also included the study of LEN as the experimental group. The control group (C) could be any immunotherapy or targeted therapy, excluding any local treatment such as hepatic arterial infusion chemotherapy or radiotherapy. The trial must be a phase III randomized controlled trial, with OS as the primary outcome and PFS, objective response rate (ORR), and treatment-related adverse events (TRAEs) disclosed. RECIST 1.1 was chosen as the reference method for PFS and ORR to allow for reproducible comparison across trials. All participants in the study were patients with unresectable or locally advanced HCC who had not undergone systemic therapy before and had no other coexisting cancers. The PubMed, MEDLINE, Embase, and Cochrane Library databases were searched from the time of U.S. approval of SOR for the treatment of uHCC (2007) to January 31st, 2024, without any language requirements. In addition, conference proceedings published by several major scientific societies were also manually searched up to January 31st, 2024, including the American Society of Clinical Oncology (ASCO), European Society of Medical Oncology (ESMO), European Association for the Study of the Liver (EASL) and American Association for the Study of Liver Diseases (AASLD). Full search strategies are provided in Table 1 and complete search terms are provided in the Supplementary material. Two independent researchers screened the records according to the preferred reporting items for systematic reviews and meta-analysis criteria and eliminated articles that did not meet the inclusion requirements by reading the title, abstract, and full text of the article. When disputes arose between two independent researchers, a third researcher was consulted.

**Table 1:** Characteristics of studies and participants included in the all-trials evidence network

**Group**	**Anti-PD-1/L1 antibody + VEGF-inhibitor**		**Anti PD-1/L1 antibody + VEGFR-targeted TKI**						**Monotherapy**
Trial, Author et al. (Year)	IMbrave150, Finn et al. (2022)	ORIENT-32, Zheng et al. (2021)	COSMIC-312, Robin et al. (2022)				CARES-310, Shu et al. (2023)	LEAP-002, Richard et al. (2023)	REFLECT, Masatoshi et al. (2018)
Network intervention (*n*)	Atezolizumab + bevacizumab (336)	Sintilimab + bevacizumab biosimilar (380)	PITT	Atezolizumab + cabozantinib (250)	ITT	Atezolizumab + cabozantinib (432)	Camrelizumab + rivoceranib (272)	LEN + pembrolizumab (395)	LEN (478)
Network comparator (*n*)	SOR (165)	SOR (191)		SOR (122)		SOR (217)	SOR (271)	LEN + placebo (399)	SOR (476)
Design	Open-label randomized, controlled trial	Open-label randomized, controlled trial	Open-label randomized, controlled trial				Open label randomized, controlled trial	Double-blind randomized, controlled trial	Open-label randomized, controlled trial
Age, mean or median (y)	I: 64; C: 66	I: 53; C: 54	I: 65; C: 64		I: 64; C: 64		I: 58; C:56	I: 66; C: 66	I: 63; C: 62
Age ≥ 65 years (%)	I: 48; C: 55	NA	NA		NA		I: 30; C:23	I: 53; C: 54	I: 44; C: 41
Sex, male (%)	I: 82; C: 83	I: 88; C: 90	I: 86; C: 88		I: 83; C: 86		I: 83; C: 85	I: 80; C: 82	I: 85; C: 84
Follow-up, median (months)	I: 17.6; C: 10.4	I: 10.0; C: 10.0	15.8		13.3		14.5	32.1	I: 17.6; C: 10.4
Region, Asia* (%)	I: 56; C: 58	I: 100; C: 100	I: 27; C: 30		I: 29; C: 33		I: 83; C: 83	I: 31; C: 31	I: 56; C: 58
ECOG PS, 0 (%)	I: 62; C: 62	I: 48; C: 48	I: 65; C: 61		I: 64; C: 66		I: 44; C: 43	I: 68; C: 68	I: 64; C: 63
ECOG PS, 1 (%)	I: 38; C: 38	I: 52; C: 52	I: 35; C: 28		I: 36; C: 34		I: 56; C: 57	I: 32; C: 32	I: 36; C: 37
Etiology, HBV (%)	I: 49; C: 46	I: 94; C: 94	I: 30; C: 29		I: 29; C: 30		I: 76; C: 73	I: 49; C: 49	I: 53; C: 48
Etiology, HCV (%)	I: 21; C: 22	I: 2; C: 4	I: 28; C: 28		I: 32; C: 31		I: 8; C: 11	I: 23; C: 22	I: 19; C: 26
Etiology, non-viral (%)	I: 30; C: 32	NA	I: 42; C: 43		I: 39; C: 40		I: 15; C: 17	I: 63; C: 59	NA
AFP ≥ 400 µg/L (%)	I: 38; C: 37	I: 43; C:42	I: 34; C: 30		I: 38; C: 30		I: 35; C: 37	I: 30; C: 33	NA
Child-Pugh, score A (%)	I: 99; C: 100	I: 96; C: 95	I: 100; C: 100		I: 100; C: 100		I: 100; C: 100	I: 99.5; C: 99.5	I: 99; C: 99
MVI (%)	I: 38; C: 43	I: 28; C: 26	I: 34; C: 31		I: 31; C: 28		I: 15; C: 19	I: 18; C: 16	I: 23; C: 19
EHS (%)	I: 63; C: 56	I: 73; C: 75	I: 54; C: 57		I: 54; C: 56		I: 64; C: 66	I: 63; C: 61	I: 61; C: 62
BCLC, grade B (%)	I: 15; C: 15	I: 15; C: 14	I: 33; C: 34		I: 32; C: 33		I: 14; C: 15	I: 22; C: 24	I: 22; C: 19
BCLC, grade C (%)	I: 82 C: 81	I: 85; C: 86	I: 67; C: 66		I: 68; C: 67		I: 86; C: 85	I: 78; C: 76	I: 78 C: 81
PD-L1 ≥ 1 (%)	I: 64; C: 57	NA	NA		NA		I: 23; C: 26	NA	NA
Prior local therapy (%)	I: 50; C: 52	I: 81; C: 85	I: 65; C: 64		I: 38; C: 39		I: 59; C: 55	I: 49; C: 51	NA
Subsequent therapy (%)	NA	NA	NA		TKI: 14; IO: 4		TKI: 27.6; IO: 14.7	TKI: 31.4; IO: 14.4	NA

Subsequent therapy is just for the intervention group. *: The study of Asia in this table does not include Japan; ITT: intention-to-treat; PITT: PFS ITT; MVI: macrovascular invasion; EHS: Extrahepatic Spread; I: intervention group; NA: not available; IO: immunotherapy; y: year old; ECOG: Eastern Cooperative Oncology Group; PS: performance status; HBV: hepatitis B viral; HCV: hepatitis C viral; AFP: alpha-fetoprotein; BCLC: Barcelona Clinic Liver Cancer

In the NMA, we chose OS as the primary endpoint to evaluate the efficacy of treatment, and PFS, OR, and the incidence of TRAEs were used to evaluate the efficacy and safety of treatment as a supplement.

The research protocol was registered in PROSPERO (registration code CRD42022375512).

### Data analysis

The relevant data extracted from the included studies was clearly showed in Table 2. OS, PFS, and ORR were used as the indicators of treatment efficacy, and their hazard ratios (HRs), odds ratios (ORs), and 95% confidence intervals (CIs) were calculated. Relative risks (RRs) and 95% CIs for the incidence of all grade TRAEs and grade ≥ 3 TRAEs were calculated to assess the safety of the treatment. A subgroup analysis for OS was performed. We believe that age, region, aetiology, MVI, and EHS are all known as prognostic factors, which may be used as the direction of subgroup discussion. Under a Bayesian framework, R software 4.2.2. (https://cran.r-project.org/) JAGS software 4.3.1 (https://sourceforge.net/projects/mcmc-jags/), “rjags” package, and “gemtc” package were used in model fitting. Non-informative priors were set, and posterior distributions were obtained using 100,000 iterations after 10,000 burns, and a thinning interval of 10. We applied three fixed knots at 25%, 50%, and 75% from the *I*^2^ test as predefined indicators of mild, moderate, and high heterogeneity, respectively. For the analysis, forest plots were drawn to directly show the comparison results. In addition, we calculated the Δ deviance information criterion (DIC: difference in deviance information criterion of inconsistent model-DIC of consistent model). The convergence of the model evaluated the potential scale reduced factor (PSRF). If PSRF is close to 1 and ΔDIC < 5, the convergence is considered favorable, and the consistency of the homogeneity model would be considered reliable enough for analysis [14]. For the test showing *I*^2^ > 50%, we calculated pooled data via a random-effects model, otherwise, we applied a fixed-effects model in the analysis. At the same time, the selection of the model is also based on ΔDIC. Moreover, we also estimated the rank probability of each treatment to represent the probability of being the best treatment, as a kind of evidence of indirect comparison. According to the Cochrane risk of bias assessment tool with a judgement of “low risk”, “unknown risk” and “high risk” in risk of bias.

**Table 2:** Outcomes of studies included in the NMA

**Trial, Author et al.** **(Year)**	**Treatments**	**Median OS, months** **(95% CI)**	**HR** **(95% CIs)**	**Median PFS, months** **(95% CI)**	**HR** **(95% CIs)**	**ORR** **(95% CI)^※^**
IMbrave150, Finn et al. (2022)	Atezolizumab + bevacizumab	19.2 (17.0–23.7)	0.66 (0.52–0.85)	6.9 (5.7–8.6)	0.65 (0.53–0.81)	30 (25–35)
	SOR	13.4 (11.4–16.9)		4.3 (4.0–5.6)		11 (7–17)
ORIENT-32, Zheng et al. (2021)	Sintilimab + bevacizumab biosimilar	NR	0.57 (0.43–0.75)	4.6 (4.1–5.7)	0.56 (0.46–0.70)	21 (17–25)
	SOR	10.4 (8.5–NR)		2.8 (2.7–3.2)		4 (2–8)
COSMIC-312, Robin et al. (2022)	Atezolizumab + cabozantinib	15.4 (96% CI 13.7–17.7)^§^	0.90 (96% CI 0.69–1.18)^§^	6.8 (99% CI 5.6–8.3)^*^	0.63 (99% CI 0.44–0.91)^*^	11 (8–14)^§^
	SOR	15.5 (96% CI 12.1–NR)^§^		4.2 (99% CI 2.8–7.0)^*^		4 (2–7)^§^
CARES-310, Shu et al. (2023)	Camrelizumab + rivoceranib	22.1 (19.1–27.2)	0.62 (0.49–0.80)	5.6 (5.5–6.3)	0.52 ( 0.41–0.65)	25 (20–31)
	SOR	15.2 (13.0–18.5)		3.7 (2.8–3.7)		6 (3–10)
LEAP-002, Richard et al. (2023)	LEN + rembrolizumab	21.2 (19.0–23.6)	0.84 (0.71–1.00)	8.2 (6.4–8.4)	0.83 (0.71–0.98)	26 (22–31)
	LEN + placebo	19.0 (17.2–21.7)		8.1 (6.3–8.3)		18 (14–22)
REFLECT, Masatoshi et al. (2018)	LEN	13.6 (12.1–14.9)	0.92 (0.79–1.06)	7.4 (6.9–8.8)	0.66 (0.57–0.77)	19 (15–22)
	SOR	12.3 (10.4–13.9)		3.7 (3.6–4.6)		7 (4–9)

^§^ According to ITT population; ^*^ according to PITT population; ^※^ IMbrave150 were investigator assessed, others were Biomedical Isotope Research Center. NR: not reach

## Results

### Screening results and basic characteristics of the included studies

**Figure 1:**
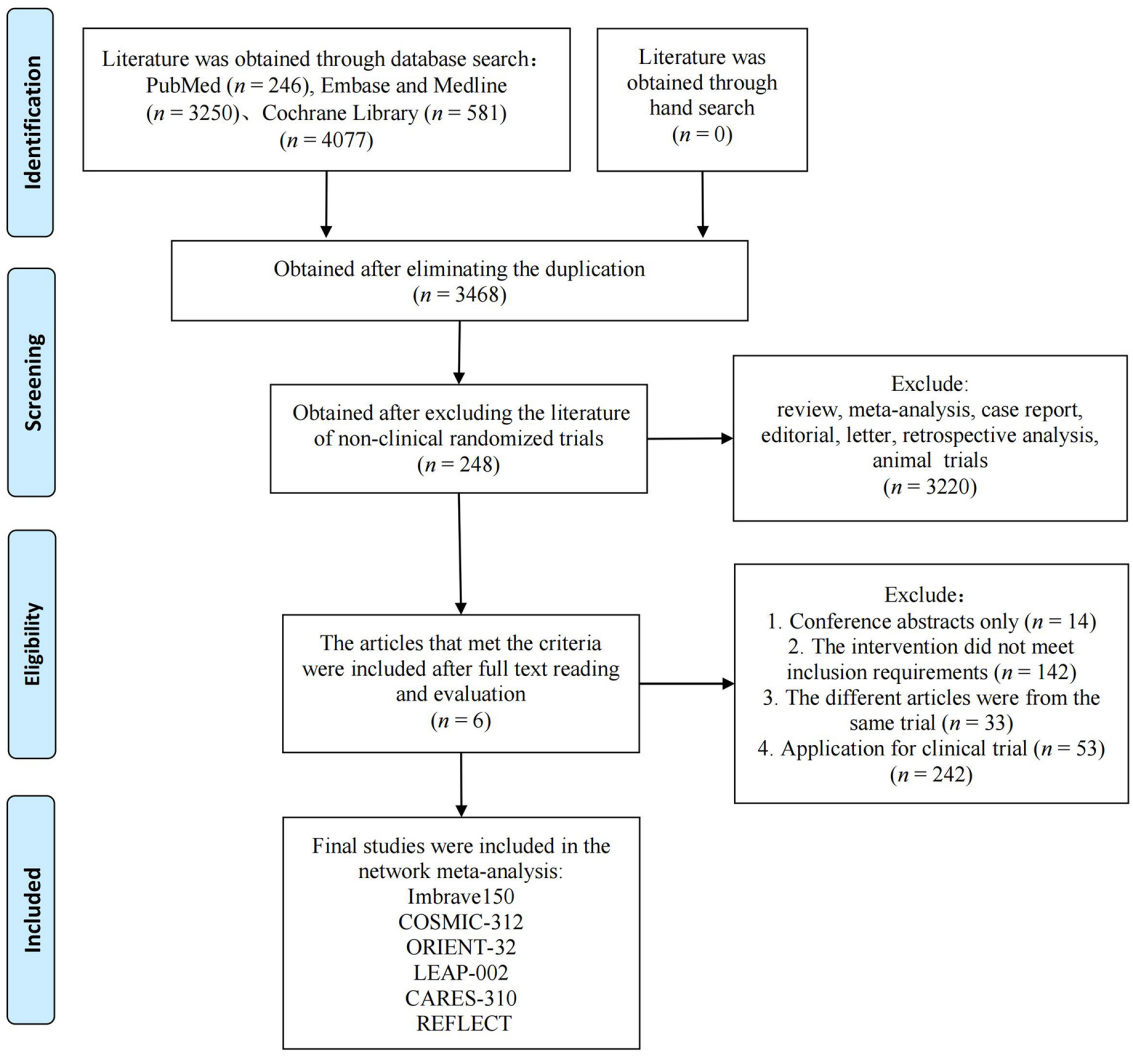
Flowchart of study selection

**Figure 2:**
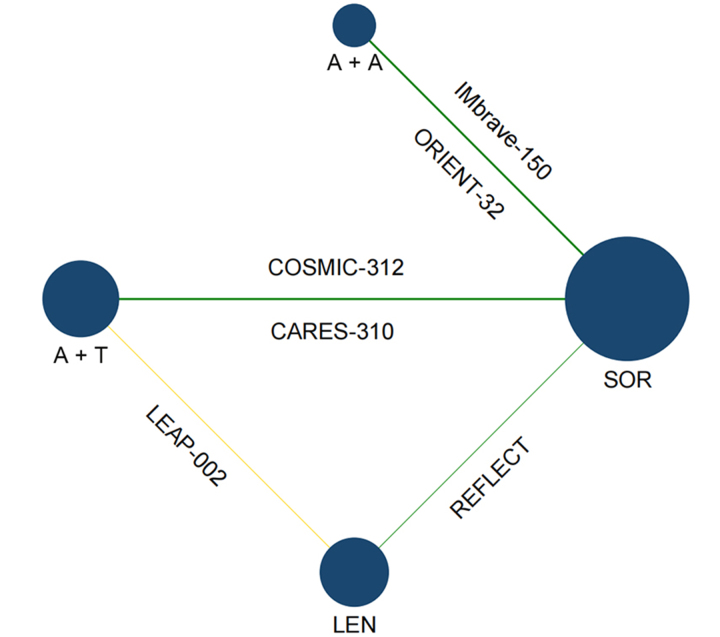
Network evidence plot for A + A *vs.* A + T

As reported in Figure 1, the 4,077 records were obtained through database search, of which 248 underwent full-text reading. A total of 242 studies were removed, and 6 were included in the NMA: IMbrave150 [15], ORIENT-32 [9], COSMIC-312 [12], LEAP-002 [11], CARES-310 [10] and REFLECT [7]. IMbrave150 and ORIENT-32 tested A + A and SOR, CARES-310 tested A + T and SOR, LEAP-002 tested A + T and LEN plus placebo, and REFLECT tested LEN and SOR. COSMIC-312 included three arms of treatment; however, since one arm (SOR) was available for comparison, only atezolizumab plus cabozantinib was considered in the NMA. A network plot of the included studies is provided in Figure 2.

Overall, 4,012 patients were included in the analysis: among them, 716 patients were treated with A + A, 1,099 patients were treated with A + T, 877 patients were treated with LEN, and 1,320 patients were treated with SOR. SOR was the control arm in all included trials, except for the LEAP-002 study, which investigated the A + T against LEN.

The characteristics of the studies and participants included in the NMA are summarized in Table 1, and the baseline characteristics of the patient populations are in part due to imbalances in the research. LEAP-002 was a double-blind trial and the others were open-label. Regarding the reporting time for trials, the median follow-up time for the experimental arm of ORIENT-32 (10.0 months) was shorter than that for the other trials: IMbrave150 (17.6 months), COSMIC-312 (15.8 months), LEAP-002 (32.1 months), CARES-310 (14.5 months) and REFLECT (17.6 months). In addition, patients in ORIENT-32 (I: 53 years; C: 54 years) and CARES-310 (I: 58 years; C: 56 years) were younger than those in other trials. There is a huge difference in patients’ regions. All patients in ORIENT-32 came from Asia, while nationalities varied among the IMbrave150 (I: 40%; C: 41%), COSMIC-312 (I: 28%; C: 29%), LEAP-002 (I: 31%; C: 31%), CARES-310 (I: 83%; C: 83%) and REFLECT (I: 67%; C: 67%) trials. Almost all patients were Child-Pugh score A, except ORIENT-32 (I: 96%; C: 95%), which is relevant to the fact that patients with both Child-Pugh A and B scores were included in ORIENT-32. In each study, a small proportion of patients had MVI, EHS, or both. Only IMbrave150 and CARES-310 reported patients’ PD-L1 status.

### Risk of bias assessment

The Cochrane Collaboration’s tool suggested that the risk of bias was generally low across all studies. In allocation concealment, blinding of outcome assessment, and selective reporting, all studies showed low risk. However, all but LEAP-002 were open label, which made blinding of participants and personnel (performance bias) the greatest concern for the included studies. Detailed information on the risk of bias assessment is provided in Figure S1.

### Results of the outcome measures

OS, PFS, ORR, and TRAEs for the included studies are summarized in Table 2.

#### Results of OS

**Figure 3:**
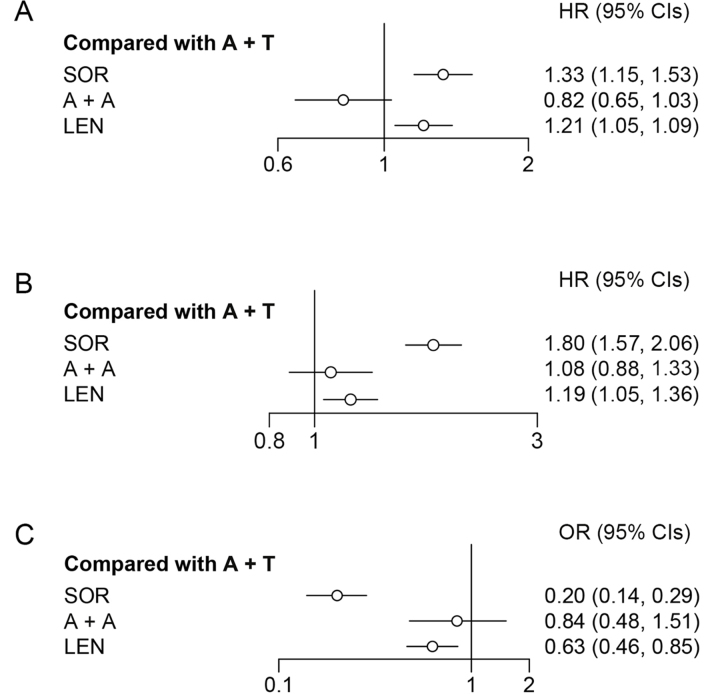
Forest plot for OS, PFS, and ORR. (A) Forest plot for OS, considering all other treatments as exposure and A + T as reference; (B) forest plot for progression free survival, considering all other treatments as exposure and A + T as reference; (C) forest plot for objective response rate, considering all other treatments as exposure and A + T as reference

According to the rank probability, which ranked all therapies for their probability of being the most effective in prolonging survival time, A + A (95%) showed better performance than A + T (5%; Table 3). Meanwhile, the fixed effects model (*I*^2^ = 37%) suggested that A + A and A + T were not significantly different (HR = 0.82, 95% CI: 0.65–1.04; Figure 3) in OS.

**Table 3:** Results of the endpoints

**Result endpoint**	**OS**	**PFS**	**ORR**	**all TRAE**	**≥ 3 TRAE**
A + A *vs.* A + T	0.82 (0.65, 1.04)	1.08 (0.88, 1.33)	0.84 (0.48, 1.51)	0.91 (0.82, 1.00)	0.65 (0.54, 0.77)
Rank probability (%)	A + A 95.28	A + T 77.64	A + T 71.87	A + A 92.62	A + A 71.08
	A + T 4.72	A + A 22.18	A + A 28.09	A + T 1.42	SOR 28.92
	LEN 0.00	LEN 0.18	LEN 0.04	LEN 4.02	A + T 0.00
	SOR 0.00	SOR 0.00	SOR 0.00	SOR 1.94	LEN 0.00
Model	FIXED	FIXED	FIXED	RANDOM	FIXED
Heterogeneity (*I*^2^%)	Medium (37)	Low (4)	Low (9)	Medium (47)	Low (22)
Consistency (ΔDIC)	2	2	2	1	3
PSRF	1	1	1	1	1

To find the source of heterogeneity and provide a direction for further research, we conducted a subgroup analysis. A + A showed better results in prolonging OS with higher rank probability overall, and it was similar in four subgroups, including region, aetiology, ECOG frequency score, and BCLC grade. However, A + T had a greater probability of prolonged survival time for patients with AFP ≥ 400, MVI, or EHS. More details are shown in Table S1.

#### Results of PFS

A fixed effects model (*I*^2^ = 4%) was used to analyze PFS. According to the rank probability for PFS (Table 3), the combination of A + T (77%) was more likely to be the most effective treatment in reducing the risk of disease progression than A + A (22%). However, there was no significant difference between A + A and A + T (HR = 1.08, 95% CI: 0.88–1.33; Figure 3).

#### Results of ORR

A + T reported a 72% probability of being the most effective therapy for objective responses, which was evaluated by a blinded independent radiology committee, followed by A + A (28%; Table 3); no significant difference was observed between A + A and A + T (OR = 0.84, 95% CI: 0.48–1.51; Figure 3).

#### Safety results

**Figure 4:**
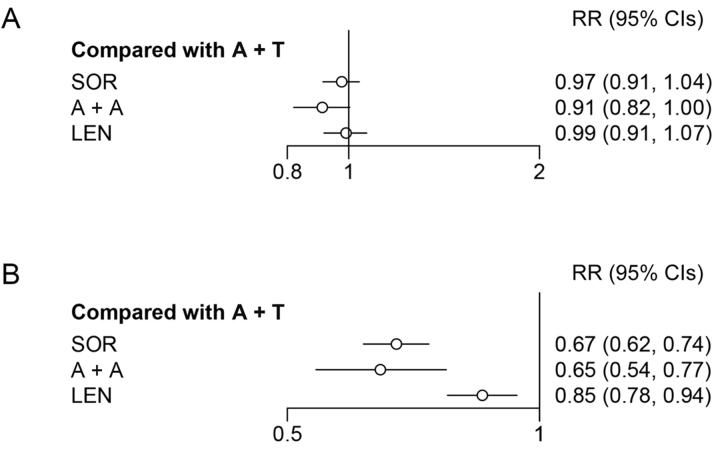
Forest plot for all grade and ≥ 3 grade TRAEs. (A) Forest plot for all grade TRAE, considering all other treatments as exposure and A + T as the reference; (B) forest plot for ≥ 3 grade TRAE, considering all other treatments as exposure and A + T as reference

TRAEs were graded according to the National Cancer Institute Common Terminology Criteria for Adverse Events version 4 (in IMbrave150 and REFLECT studies) and 5 (in COSMIC-312 and ORIENT-32 studies). The criteria were not clear in LEAP-002 and CARES-310. A + A was safer than A + T for both all grade TRAEs and particularly for ≥ 3 grade TRAEs (RR = 0.91, 95% CI: 0.82–1.00; RR = 0.65, 95% CI: 0.54–0.77; Figure 4). Platelet count decreased, hypertension, hypothyroidism, diarrhoea, and PPE syndrome were common AEs and were reported in all included studies. More data on security is provided in Table S2 and Table S3.

## Discussion

To our knowledge, this is the first NMA to evaluate the clinical efficacy and safety of combining two combination regimens in patients with unresectable or locally advanced HCC as the first-line treatment [16, 17]. The VEGF antibodies included in our study were bevacizumab and its biosimilar, which target VEGF-A and act by inhibiting the biological activity of human VEGF. However, TKIs participating in this NMA include SOR and LEN, whose targets include fibroblast growth factor receptor, platelet-derived growth factor receptor, and the main target VEGFR. Although only a few studies were included in this study, the six included studies were all high-quality randomized controlled studies with a low risk of bias and similar baseline characteristics of patients.

This study showed anti-PD-1/L1 antibody combined with VEGF-antibody appeared to be the most effective therapeutic strategy in reducing the risk of death, while anti-PD-1/L1 antibody combined with VEGFR-targeted TKI was more likely to bring longer PFS and higher ORR in patients at the cost of lower safety rate.

In this study, OS was chosen as the primary endpoint, which could provide the most objective clinical assessment of efficacy for cross-trial comparison. The results showed that there was no significant difference in OS between the two treatment regimens, which was similar to the results of a recent retrospective study [18]. In the subgroup analysis, the clinical efficacy results were generally consistent with the primary results of patient analyses based on geographic region, etiology, ECOG score, and BCLC grade. A + T showed a better ability to address MVI and EHS. Compared with those without MVI, HCC with MVI is usually characterized by worsening liver function, vulnerability to blood metastasis, a higher incidence of complications associated with portal hypertension, and intolerance to treatment [19]. Patients with EHS usually have a poor prognosis, but the specific situation is closely related to the site of distant metastasis. Peritoneal metastasis and bile duct involvement in HCC patients treated with Immune checkpoint inhibitors may indicate significantly poor OS but have no significant impact on the prognosis of ORR [20]. In a prospective trial of LEN plus pembrolizumab, EHS was not an independent risk factor for OS, which may be related to front-line therapy in some patients [21]. Thus, we do not deny that the inadequacy of the included studies has contributed to the error in the results, so more high-quality trials should be promoted.

Different from the results of OS, A + T had a better effect in prolonging patients’ PFS and reducing the size of solid tumours. The results are consistent with previous clinical studies. A pilot study involving 10 HCC patients with vascular infiltration showed that after treatment with anti-PD-1 antibody combined with VEGFR-targeted TKI, 7 patients achieved PR, 3 patients achieved CR, and all 10 patients underwent surgery with a recurrence-free survival rate of 75% within 12 months [22]. Another study also indicated that this conversion therapy strategy can be effective and safe for hepatectomy after careful preparation and patient evaluation [23]. At the same time, A + T combination treatment of renal cell carcinoma and non-small cell lung cancer also has an excellent tumour reduction effect with a high ORR [24, 25].

A + T showed higher toxicity with significantly higher incidences of all grade TRAEs and grade ≥ 3 TRAEs than A + A. As an important organ of drug metabolism, the liver is one of the most susceptible organs to cancer immunotherapy, and the liver damage caused by immunotherapy is often referred to as immune-mediated hepatitis (IMH) and often can be diagnosed by the increase in abnormally elevated serum liver enzymes [26]. The rate of abnormal elevation of serum liver enzymes in patients in the A + T group was higher than that in the A + A group (Table S3). On the other hand, it reflected that A + T might effectively improve the effect of immunotherapy as overactivity of T cells may lead to the destruction of immune tolerance in the liver, making it susceptible to acute inflammation and hepatitis [27, 28]. To control IMH, the guidelines recommend a detailed examination of the patient's liver condition prior to the use of the drug, cessation of immunotherapy as soon as IMH occurs, and the use of corticosteroids as treatment agents [29–31]. In the A + A group, the most common TRAEs were hypertension and proteinuria, which were closely related to the nephrotoxicity caused by VEGF antibodies [32, 33]. For the sake of patient safety, if patients suffer from complications such as diabetes or kidney disease, comprehensive renal function evaluation should be performed before A + A therapy, and patients’ hypertension must be well controlled [33].

This NMA also has limitations that need further consideration and exploration. First, among the included trials, the data of two trials were obtained from the conference presentation, and the data were incomplete, which would eventually lead to the deviation of the estimation of the relative effect. In addition, the varied follow-up time among the trials and the open-label nature of one included trial may have introduced bias. Hence, more high-quality prospective studies are needed to better evaluate the optimal treatment. Second, a meta-analysis found that the Child-Pugh score was subjective to a certain extent, and the Albumin Bilirubin score could better stratify patients [34]. Therefore, how to conduct more accurate stratification and precise treatment for patients before the implementation of treatment may be a major direction of our future research. Third, a recent study showed that discontinuation of A + T in the absence of other therapeutic adjustments was associated with poor outcomes [35]. However, the included studies did not mention drug dose changes and discontinuation in detail, and only three studies mentioned the use of TKIs for subsequent maintenance therapy (LEAP-002: 34.9%; COSMIC-312: 14%; CARES-310: 29.8%), which prevented us from exploring this aspect further. Thus, the search for the best follow-up regimen or drug use sequence will become an important challenge in the management of HCC.

In conclusion, we suggested that A + A had the greatest probability of delivering the longest OS, while A + T was correlated with larger PFS benefit at the cost of a lower safety rate.

## Abbreviations

A + A: anti-programmed death 1/ligand-1 antibody + anti-vascular endothelial growth factor antibody
A + T: anti-programmed death 1/ligand-1 antibody + anti-vascular endothelial growth factor receptor-targeted tyrosine kinase inhibitor
BCLC: Barcelona Clinic Liver Cancer
C: control group
CIs: confidence intervals
DIC: deviance information criterion
ECOG: Eastern Cooperative Oncology Group
EHS: extrahepatic spread
HCC: hepatocellular carcinoma
HR: hazard ratio
I: intervention group
IMH: immune-mediated hepatitis
MVI: macrovascular invasion
NMA: network meta-analysis
ORR: objective response rate
OS: overall survival
PD-1/L1: programmed death 1/ligand-1
PFS: progression-free survival
PSRF: potential scale reduced factor
RR: relative risk
TKI: tyrosine kinase inhibitor
TRAEs: treatment-related adverse events
uHCC: unresectable hepatocellular carcinoma
VEGF: vascular endothelial growth factor
VEGFR: vascular endothelial growth factor receptor

## Supplementary materials

The supplementary methods for this article is available at: https://www.explorationpub.com/uploads/Article/file/1002236_sup_1.pdf.

## Declarations

### Availability of data and materials

All data generated or analysed for this study are included in the manuscript and the supplementary files.

### Copyright

© The Author(s) 2024.
