# Supplementary material for: Anti-PD-1/L1 antibody plus anti-VEGF antibody vs. plus VEGFR-targeted TKI as first-line therapy for unresectable hepatocellular carcinoma: a network meta-analysis
Source: Explor Target Antitumor Ther. 2024 Jun 17;5(3):568–80. doi: 10.37349/etat.2024.00236 (PMC11220314; doi:10.37349/etat.2024.00236)
Supplement: Supplementary file 1 [file 1002236_sup_1.pdf]

Supplementary Method: Search strategy for PubMed

(((((Liver[MeSH Terms]) AND (Neoplasms[MeSH Terms])) OR (((Liver Neoplasms[MeSH Terms]) OR (Adenoma, Liver Cell[MeSH Terms])) OR (Carcinoma, Hepatocellular[MeSH Terms]))) OR ((hepatoma[Title/Abstract] OR HCC[Title/Abstract]) OR ((liver[Title/Abstract] OR Hepatic[Title/Abstract] OR hepatocellular[Title/Abstract]) AND (cancer[Title/Abstract] OR carcinoma[Title/Abstract] OR Neoplasm[Title/Abstract] OR tumor[Title/Abstract] OR tumour[Title/Abstract] OR malignant[Title/Abstract])))) AND (((((Bevacizumab[MeSH Terms]) OR (Bevacizumab[Title/Abstract] OR Avastin[Title/Abstract])) OR ((Ramucirumab[Supplementary Concept]) OR (Ramucirumab[Title/Abstract] OR Cyramza[Title/Abstract]))) AND (((Immune Checkpoint Inhibitors[MeSH Terms] OR Immunosuppressive Agents[MeSH Terms]) OR (Immunosuppress*[Title/Abstract] OR immune checkpoint inhibitors[Title/Abstract] OR ICIs[Title/Abstract] OR anti-programmed cell death protein 1[Title/Abstract] OR anti-PD-1 antibody[Title/Abstract] OR anti-Programmed cell death ligand 1[Title/Abstract] OR anti-PD-L1 antibody[Title/Abstract] OR Programmed Death 1[Title/Abstract] OR Programmed Death Ligand 1[Title/Abstract] OR PD-1[Title/Abstract] OR PD1[Title/Abstract] OR PDL1[Title/Abstract] OR PD-L1[Title/Abstract])) OR ((((((((((((((((Nivolumab[MeSH Terms]) OR (Nivolumab[Title/Abstract] OR OPDIVO[Title/Abstract])) OR ((Pembrolizumab[Supplementary Concept]) OR (Pembrolizumab[Title/Abstract] OR Keytruda[Title/Abstract]))) OR ((Sintilimab[MeSH Terms]) OR (Sintilimab[Title/Abstract] OR Tyvyt[Title/Abstract]))) OR ((Camrelizumab[Supplementary Concept]) OR (Camrelizumab[Title/Abstract] OR AiRuiKa[Title/Abstract]))) OR ((Tislelizumab[Supplementary Concept]) OR (Tislelizumab[Title/Abstract] OR BGB-A317[Title/Abstract]))) OR ((Toripalimab[Supplementary Concept]) OR (Toripalimab[Title/Abstract] OR Tuoyi[Title/Abstract]))) OR ((Cemiplimab[Supplementary Concept]) OR (Libtayo[Title/Abstract] OR Cemiplimab[Title/Abstract]))) OR ((Spartalizumab[Supplementary Concept]) OR (PDR001[Title/Abstract] OR Spartalizumab[Title/Abstract]))) OR ((Zimberelimab[Supplementary Concept]) OR (Zimberelimab[Title/Abstract] OR GLS-010[Title/Abstract]))) OR ((Dostarlimab[Supplementary Concept]) OR (Jemperli[Title/Abstract] OR Dostarlimab[Title/Abstract]))) OR ((Penpulimab[Supplementary Concept]) OR (Penpulimab[Title/Abstract]))) OR

((Durvalumab[Supplementary Concept]) OR (Durvalumab[Title/Abstract] OR Imfinzi[Title/Abstract]))) OR ((Atezolizumab[Supplementary Concept]) OR (Atezolizumab[Title/Abstract] OR Tecentriq[Title/Abstract]))) OR ((Envafolimab[Supplementary Concept]) OR (Envafolimab[Title/Abstract] OR KN035[Title/Abstract]))) OR ((Sugemalimab[Supplementary Concept]) OR (Sugemalimab[Title/Abstract] OR CS1001[Title/Abstract]))) OR (Prolgolimab[Title/Abstract] OR Serplulimab[Title/Abstract])))) OR ((((Immune Checkpoint Inhibitors[MeSH Terms] OR Immunosuppressive Agents[MeSH Terms]) OR (Immunosuppress*[Title/Abstract] OR immune checkpoint inhibitors[Title/Abstract] OR ICIs[Title/Abstract] OR anti-programmed cell death protein 1[Title/Abstract] OR anti-PD-1 antibody[Title/Abstract] OR anti-Programmed cell death ligand 1[Title/Abstract] OR anti-PD-L1 antibody[Title/Abstract] OR Programmed Death 1[Title/Abstract] OR Programmed Death Ligand 1[Title/Abstract] OR PD-1[Title/Abstract] OR PD1[Title/Abstract] OR PDL1[Title/Abstract] OR PD-L1[Title/Abstract])) OR ((((((((((((((((Nivolumab[MeSH Terms]) OR (Nivolumab[Title/Abstract] OR OPDIVO[Title/Abstract])) OR ((Pembrolizumab[Supplementary Concept]) OR (Pembrolizumab[Title/Abstract] OR Keytruda[Title/Abstract]))) OR ((Sintilimab[MeSH Terms]) OR (Sintilimab[Title/Abstract] OR Tyvyt[Title/Abstract]))) OR ((Camrelizumab[Supplementary Concept]) OR (Camrelizumab[Title/Abstract] OR AiRuiKa[Title/Abstract]))) OR ((Tislelizumab[Supplementary Concept]) OR (Tislelizumab[Title/Abstract] OR BGB-A317[Title/Abstract]))) OR ((Toripalimab[Supplementary Concept]) OR (Toripalimab[Title/Abstract] OR Tuoyi[Title/Abstract]))) OR ((Cemiplimab[Supplementary Concept]) OR (Libtayo[Title/Abstract] OR Cemiplimab[Title/Abstract]))) OR ((Spartalizumab[Supplementary Concept]) OR (PDR001[Title/Abstract] OR Spartalizumab[Title/Abstract]))) OR ((Zimberelimab[Supplementary Concept]) OR (Zimberelimab[Title/Abstract] OR GLS-010[Title/Abstract]))) OR ((Dostarlimab[Supplementary Concept]) OR (Jemperli[Title/Abstract] OR Dostarlimab[Title/Abstract]))) OR ((Penpulimab[Supplementary Concept]) OR (Penpulimab[Title/Abstract]))) OR ((Durvalumab[Supplementary Concept]) OR (Durvalumab[Title/Abstract] OR Imfinzi[Title/Abstract]))) OR ((Atezolizumab[Supplementary Concept]) OR (Atezolizumab[Title/Abstract] OR Tecentriq[Title/Abstract]))) OR ((Envafolimab[Supplementary Concept]) OR (Envafolimab[Title/Abstract] OR

KN035[Title/Abstract]))) OR ((Sugemalimab[Supplementary Concept]) OR (Sugemalimab[Title/Abstract] OR CS1001[Title/Abstract]))) OR (Prolgolimab[Title/Abstract] OR Serplulimab[Title/Abstract]))) AND ((((Protein Kinase Inhibitors[MeSH Terms])) OR (Tyrosine kinase inhibitor[Title/Abstract] OR TKI[Title/Abstract])) OR ((((((((((((((((Sorafenib[MeSH Terms]) OR (Sorafenib[Title/Abstract] OR Nexavar[Title/Abstract])) OR ((Lenvatinib[Supplementary Concept]) OR (Lenvima[Title/Abstract] OR Lenvatinib[Title/Abstract]))) OR ((Cabozantinib[Supplementary Concept]) OR (Cabozantinib[Title/Abstract] OR Cometriq[Title/Abstract] OR Cabometyx[Title/Abstract]))) OR ((Regorafenib[Supplementary Concept]) OR (Stivarga[Title/Abstract] OR Regorafenib[Title/Abstract]))) OR ((Axitinib[MeSH Terms]) OR (Bavencio[Title/Abstract] OR Axitinib[Title/Abstract]))) OR ((Sunitinib[MeSH Terms]) OR (Sunitinib[Title/Abstract] OR Sutent[Title/Abstract]))) OR ((Vandetanib[Supplementary Concept]) OR (Vandetanib[Title/Abstract] OR Caprelsa[Title/Abstract]))) OR ((Pazopanib[Supplementary Concept]) OR (Pazopanib[Title/Abstract] OR Votrient[Title/Abstract]))) OR ((Anlotinib[Supplementary Concept]) OR (Anlotinib[Title/Abstract] OR AL3818[Title/Abstract] OR Catequentinib[Title/Abstract]))) OR ((HMPL-013 [Supplementary Concept]) OR (HMPL-013[Title/Abstract] OR Fruquintinib[Title/Abstract]))) OR ((Apatinib[Supplementary Concept]) OR (Apatinib[Title/Abstract] OR rivoceranib[Title/Abstract]))) OR ((Surufatinib[Supplementary Concept]) OR (Surufatinib[Title/Abstract] OR HMPL-012[Title/Abstract] OR sulfatinib[Title/Abstract]))) OR ((Famitinib[Supplementary Concept]) OR (Famitinib[Title/Abstract] OR SHR1020[Title/Abstract]))) OR ((Donafenib[Supplementary Concept]) OR (Donafenib[Title/Abstract] OR Zepsun[Title/Abstract] OR CM-4307[Title/Abstract]))) OR ((Cediranib[Supplementary Concept]) OR (Cediranib[Title/Abstract] OR CS-377[Title/Abstract]))))))) AND ((((((((randomized controlled trial[Publication Type]) OR controlled clinical trial[Publication Type]) OR randomized[Title/Abstract]) OR placebo[Title/Abstract]) OR randomly[Title/Abstract]) OR "Clinical Trials as Topic"[Mesh:NoExp]) OR trial[Title])) NOT ((((animals[MeSH Terms]) NOT ((humans[MeSH Terms]) AND animals[MeSH Terms])))))

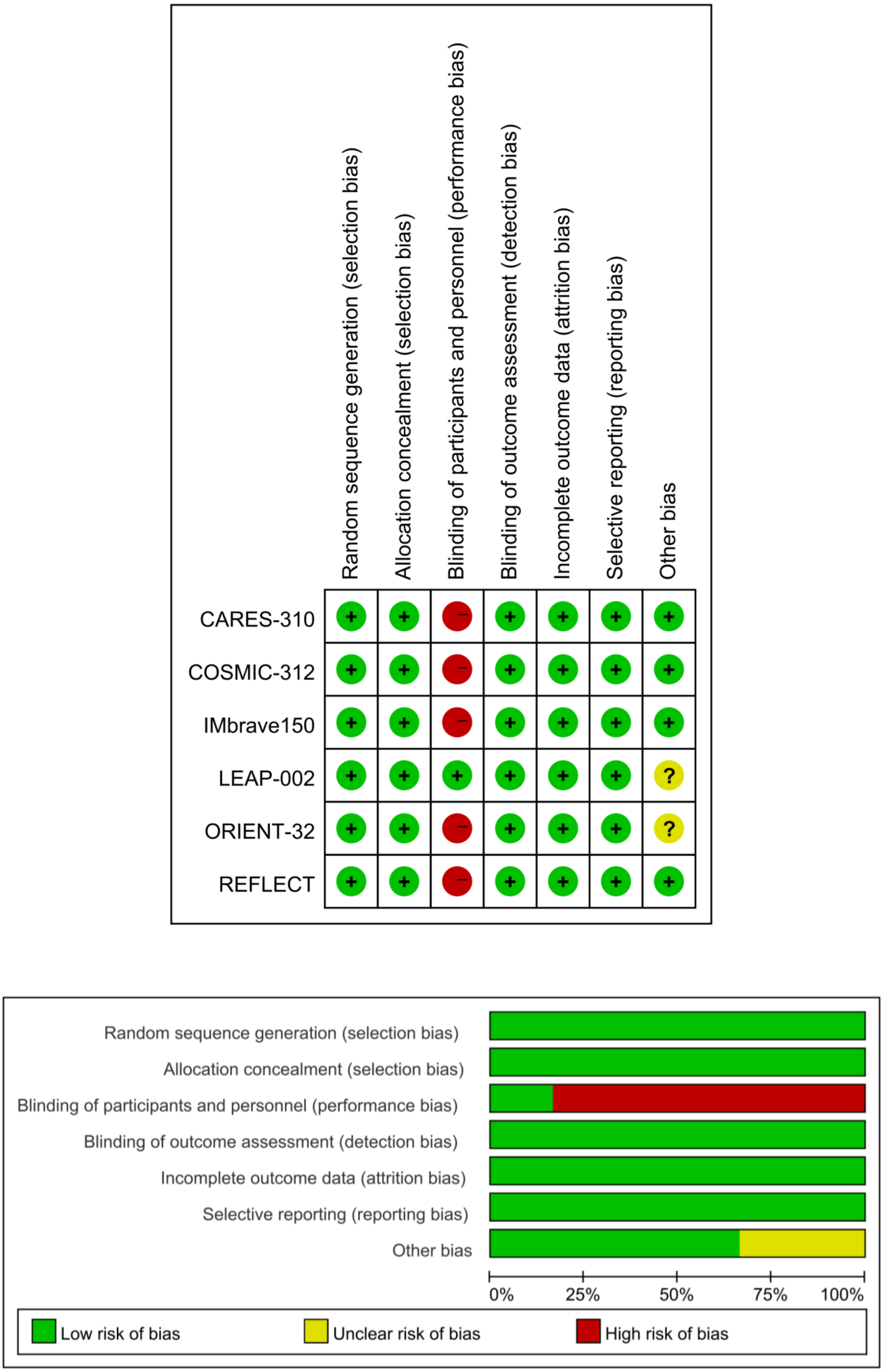

Figure S1. Assessment of methodological quality according to the Quality Assessment of Diagnostic Accuracy Studies–2 (QUADAS-2).

Table S1: Subgroup analysis of overall survival.

subgroup analysis	Region		AFP		MVI		EHS		Etiology		ECOG		BCLC	
	Asia	Other	< 400	≥ 400	(+)	(-)	(+)	(-)	HBV	HCV	PS=0	PS=1	B	C
Included trials	1；3；4；5；6		1；2；4；5		1；2；4；5		1；2；4；5		1；2；3；4；5；6	1；3；4；5；6	1；2；4；5；6		1；2；4；5；6	
A+A vs. A+T	0.92 (0.59,1.43)	0.82 (0.26,2.73)	1.03 (0.67,1.58)	1.06 (0.68,1.65)	1.13 (0.60,2.13)	0.89 (0.62,1.30)	1.04 (0.72,1.51)	0.87 (0.50,1.52)	0.93 (0.69,1.25)	0.91 (0.70,1.20)	0.94 (0.67,1.34)	0.84 (0.59,1.19)	0.46 (0.09,3.02)	0.92 (0.70,1.20)
Rank Probability (%)	A+A	A+A	A+A	**A+T**	**A+T**	A+A	**A+T**	A+A	A+A	A+A	A+A	A+A	A+A	A+A
	64.46	62.27	41.03	**60.90**	**58.62**	72.44	**57.97**	62.09	68.77	74.26	62.47	83.21	80.20	73.85
	A+T	A+T	A+T	A+A	A+A	A+T	A+A	LEN	A+T	A+T	A+T	A+T	A+T	A+T
	35.35	30.74	37.96	38.91	34.52	26.55	41.34	19.19	31.21	25.74	37.34	16.77	12.66	26.15
	LEN	SOR	LEN	LEN	LEN	LEN	LEN	A+T	LEN	LEN	LEN	LEN	LEN	LEN
	0.19	4.79	20.01	0.19	6.86	1.01	0.69	18.08	0.02	0.00	0.19	0.02	6.18	0.00
	SOR	LEN	SOR	SOR	SOR	SOR	SOR	SOR	SOR	SOR	SOR	SOR	SOR	SOR
	0.00	2.20	0.00	0.00	0.00	0.00	0.00	0.64	0.00	0.00	0.00	0.00	0.96	0.00

Trials: 1-IMbrave150, 2-ORIENT32, 3-COSMIC312, 4-CARES310, 5-LEAP002, 6-REFLECT.

Table S2: Treatment-related adverse events of studies in the network Meta-analysis.

Group	A+A						A+T				Monotherapy	
Trial, Author et al (Year)	IMbrave150, Finn et al (2022)		ORIENT-32, Zheng et al (2021)		COSMIC-312, Robin et al (2022)		CARES-310 Shu et al (2023)		LEAP-002, Richard et al (2023)		REFLECT, Masatoshi et al(2018)	
Treatment (n)	Atezolizumab+ Bevacizumab (329)	Sor (156)	Sintilimab + Bevacizumab biosimilar (380)	Sor (185)	Atezolizumab+ Cabozantinib (429)	Sor (207)	Camrelizumab +Rivoceranib (272)	Sor (269)	Lenvatinib + Pembrolizumab (395)	Len (395)	Len (476)	Sor(475)
Treatment-related all Grade AE (n)	284 (86%)	148 (95%)	337 (89%)	173 (94%)	399 (93%)	186 (90%)	265 (97%)	249 (93%)	381 (97%)	378 (96%)	447(94%)	452(95%)
Treatment-related Grade ≥3 AE (n)	149 (45%)	73 (47%)	131 (34%)	68 (37%)	236 (55%)	68 (33%)	220 (81%)	141 (52%)	247 (63%)	227 (57%)	270(57%)	231(49%)
TEAE leading to discontinuation of any study treatment	NA	NA	52 (14%)	11 (6%)	84 (20%)	16 (8%)	66 (24%)	12 (5%)	71 (18%)	42 (11%)	NA	NA

Table S3: Descriptive incidence of treatments related adverse events according to NCTCA.

Trial, Author et al (Year)	**IMbrave150, Finn et al (2022)**		**ORIENT-32, Zheng et al (2021)**		**COSMIC-312, Robin et al (2022)**		**CARES-310, Shu et al (2023)**		**LEAP-002, Richard et al (2023)**		**REFLECT, Masatoshi et al (2018)**	
Treatment (n)	Atezolizumab + Bevacizumab (329)	Sorafenib (156)	Sintilimab + Bevacizumab biosimilar (380)	Sorafenib (185)	Atezolizumab + Cabozantinib (429)	Sorafenib (207)	Camrelizumab + Rivoceranib (272)	Sorafenib (269)	Lenvatinib + Pembrolizumab (395)	Lenvatinib + placebo (395)	Lenvatinib (476)	Sorafenib (475)
TRAEs (all Grade)	284 (86%)	148 (95%)	337 (89%)	173 (94%)	399 (93%)	186 (90%)	265 (97%)	249 (93%)	381 (97%)	378 (96%)	447 (94%)	452 (95%)
TRAEs (≥ 3 Grade)	149 (45%)	73 (47%)	131 (34%)	68 (37%)	236 (55%)	68 (33%)	220 (81%)	141 (52%)	247 (63%)	227 (57%)	270 (57%)	231 (49%)
Proteinuria (all Grade)	95 (29%)	8 (5%)	154 (41%)	27 (15%)	28 (7%)	12 (6%)	134 (49%)	72 (27%)	121 (31%)	138 (35%)	NA	NA
Proteinuria (≥ 3 Grade)	3 (4%)	1 (<1%)	18 (5%)	2 (1%)	7 (2%)	0	16 (6%)	5 (2%)	26 (7%)	22 (6%)	NA	NA
AST increased (all Grade)	54 (16%)	12 (8%)	81 (21%)	42 (23%)	92 (21%)	17 (8%)	147 (54%)	99 (37%)	87 (22%)	61 (15%)	NA	NA
AST increased (≥ 3 Grade)	17 (5%)	5 (3%)	4 (1%)	5 (3%)	28 (7%)	5 (2%)	45 (17%)	14 (5%)	27 (7%)	17 (4%)	NA	NA
ALT increased (all Grade)	40 (12%)	4 (3%)	63 (17%)	35 (19%)	96 (22%)	12 (6%)	127 (47%)	80 (30%)	76 (19%)	59 (5%)	NA	NA
ALT increased (≥ 3 Grade)	8 (2%)	0	4 (1%)	2 (1%)	27 (6%)	4 (2%)	35 (13%)	8 (3%)	19 (5%)	12 (3%)	NA	NA
Platelet count decreased	34 (10%)	15 (10%)	99 (26%)	40 (22%)	46 (11%)	13 (6%)	126 (46%)	89 (33%)	83 (21%)	83 (21%)	87 (18%)	58 (12%)

(all Grade)												
Platelet count decreased (≥ 3 Grade)	9 (3%)	1 (<1%)	22 (6%)	2 (1%)	8 (2%)	2 (1%)	32 (12%)	4 (2%)	17 (5%)	17 (4%)	26 (5%)	16 (3%)
Hypertension (all Grade)	93 (28%)	31 (20%)	108 (28%)	27 (15%)	82 (19%)	32 (15%)	189 (70%)	116 (43%)	171 (43%)	185 (47%)	201 (42%)	144 (30%)
Hypertension (≥ 3 Grade)	39 (12%)	14 (9%)	48 (13%)	9 (5%)	30 (7%)	13 (6%)	102 (38%)	40 (15%)	69 (17%)	68 (17%)	111 (23%)	68 (14%)
Hypothyroidism (all Grade)	33 (10%)	2 (1%)	47 (12%)	11 (6%)	83 (19%)	4 (2%)	58 (21%)	16 (6%)	158 (40%)	141 (36%)	78 (16%)	8 (2%)
Hypothyroidism (≥ 3 Grade)	0	0	0	0	0	0	0	0	1 (<1%)	0	0	0
Diarrhoea (all Grade)	36 (11%)	68 (44%)	24 (6%)	63 (34%)	181 (42%)	87 (42%)	83 (31%)	105 (39%)	159 (40%)	134 (34%)	184 (39%)	220 (46%)
Diarrhoea (≥ 3 Grade)	3 (1%)	6 (4%)	2 (<1%)	3 (2%)	15 (3%)	2 (1%)	6 (2%)	14 (5%)	25 (6%)	15 (4%)	20 (4%)	20 (4%)
PPE syndrome (all Grade)	5 (2%)	75 (48%)	1 (<1%)	102 (55%)	181 (42%)	92 (44%)	102 (38%)	163 (61%)	131 (33%)	121 (31%)	128 (27%)	249 (52%)
PPE syndrome (≥ 3 Grade)	0	13 (8%)	0	22 (12%)	34 (8%)	17 (8%)	33 (12%)	41 (15%)	19 (5%)	13 (3%)	14 (3%)	54 (11%)

AST=aspartate aminotransferase; ALT=alanine aminotransferase; PPE=palmar-plantar erythrodysaesthesia. NA= unavailable.
